# Healthy Aging Alters the Functional Connectivity of Creative Cognition in the Default Mode Network and Cerebellar Network

**DOI:** 10.3389/fnagi.2021.607988

**Published:** 2021-02-18

**Authors:** Abhishek Uday Patil, Deepa Madathil, Chih-Mao Huang

**Affiliations:** ^1^Department of Sensor and Biomedical Technology, School of Electronics Engineering, Vellore Institute of Technology, Vellore, India; ^2^Department of Biological Science and Technology, National Chiao Tung University, Hsinchu, Taiwan; ^3^Center for Intelligent Drug Systems and Smart Bio-Devices (IDS^2^B), National Chiao Tung University, Hsinchu, Taiwan; ^4^Cognitive Neuroscience Laboratory, Institute of Linguistics, Academia Sinica, Taipei, Taiwan

**Keywords:** cognitive aging, resting-state fMRI, creative cognition, functional connectivity, group independent component analysis

## Abstract

Creativity is a higher-order neurocognitive process that produces unusual and unique thoughts. Behavioral and neuroimaging studies of younger adults have revealed that creative performance is the product of dynamic and spontaneous processes involving multiple cognitive functions and interactions between large-scale brain networks, including the default mode network (DMN), fronto-parietal executive control network (ECN), and salience network (SN). In this resting-state functional magnetic resonance imaging (rs-fMRI) study, group independent component analysis (group-ICA) and resting state functional connectivity (RSFC) measures were applied to examine whether and how various functional connected networks of the creative brain, particularly the default-executive and cerebro-cerebellar networks, are altered with advancing age. The group-ICA approach identified 11 major brain networks across age groups that reflected age-invariant resting-state networks. Compared with older adults, younger adults exhibited more specific and widespread dorsal network and sensorimotor network connectivity within and between the DMN, fronto-parietal ECN, and visual, auditory, and cerebellar networks associated with creativity. This outcome suggests age-specific changes in the functional connected network, particularly in the default-executive and cerebro-cerebellar networks. Our connectivity data further elucidate the critical roles of the cerebellum and cerebro-cerebellar connectivity in creativity in older adults. Furthermore, our findings provide evidence supporting the default-executive coupling hypothesis of aging and novel insights into the interactions of cerebro-cerebellar networks with creative cognition in older adults, which suggest alterations in the cognitive processes of the creative aging brain.

## 1. Introduction

Creative thinking, defined as the iterative processes by which unusual and useful approaches to problem solving are developed, involves the dynamic integration of multiple cognitive mechanisms, including internally focused attention, proponent-response inhibitory control, and goal-directed memory retrieval (Benedek et al., 2014c, 2016; Beaty et al., 2016, 2019). The behavioral performance of this form of higher-level cognitive ability has been associated with individual variations in verbal and visuospatial working memory, crystallized and fluid intelligence, episodic memory, semantic knowledge, and executive function (Markman and Dietrich, 2010; Duff et al., 2013; Jauk et al., 2013; Silvia et al., 2013; Benedek et al., 2014b; Kenett et al., 2014; Mammarella et al., 2014; Addis et al., 2016). These associations suggest that various brain regions and networks that support multidimensional cognitive processes contribute to creative thinking.

Early neuroimaging studies identified associations between the neural substrates of creative cognition and a set of predominantly left-hemispheric and distributed brain regions, including the lateral prefrontal cortex (PFC), posterior parietal cortex (PPC), lateral temporal gyrus, and hippocampus (den Heuvel et al., 2010; Green et al., 2015; Kenett et al., 2015; Saggar et al., 2015; Ogawa et al., 2018; Chen et al., 2019; Wertz et al., 2020). Recent functional neuroimaging research in the field of network neuroscience used task-related and resting-state functional magnetic resonance imaging (fMRI) techniques to map the contributions of large-scale brain networks to various cognitive mechanisms associated with creativity (Bressler and Menon, 2010; Spreng et al., 2013; Beaty et al., 2016, 2018b, 2019). The converging fMRI evidence from these studies identified the contributions of specific patterns of functional connectivity between the default mode network (DMN), fronto-parietal executive control network (ECN), salience network (SN), visual network (VN), and lateral and medial temporal lobes to self-generated and goal-directed creative cognition processes across a range of creativity domains (Beckmann and Smith, 2005; Fox and Raichle, 2007; Fink et al., 2009, 2010, 2014; Raichle, 2010; Takeuchi et al., 2011; Abraham et al., 2012; Beaty et al., 2014, 2015, 2016, 2018a; Benedek et al., 2014a,b; Wei et al., 2014; Liu et al., 2015; Buckner and DiNicola, 2019). In a resting-state fMRI (rs-fMRI) study, Wei et al. (2014) used functional connectivity measures to investigate the critical role of the DMN in divergent thinking, and identified an association between connectivity within the DMN and the behavioral performance of divergent thinking. The authors further demonstrated that an increase in functional connectivity between the medial pre-frontal cortex and the middle temporal gyrus was associated with the processes of cognition control during divergent thinking (Wei et al., 2014). Recently, Feng et al. (2019) reported that the performance of verbal creativity was associated with a dynamic re-organization of the brain network involving various neurocognitive processes, including cognitive control, episodic memory retrieval, and imagination, and demonstrated that network interactions between the DMN and cerebellum modulate creativity in the verbal domain (Feng et al., 2019). These compelling findings support the potential contributions of large-scale brain network interactions between creativity-associated cognitive processes to individual differences in creative performance (Dietrich and Kanso, 2010; Takeuchi et al., 2012; Jung et al., 2013; Beaty et al., 2014, 2019; Wei et al., 2014).

Behavioral and neuroimaging studies conducted in younger adults have identified creative cognition as the product of dynamic and spontaneous processes involving multiple cognitive functions and large-scale brain network interactions. However, little is known about the potential effects of healthy aging on these creativity-related neurocognitive processes. Behavioral studies pertaining to age-related changes in creative cognition changes have produced conflicting evidence. A few studies have reported age-related declines in creative thought generation, whereas other studies have demonstrated that creativity performance is preserved across the human lifespan (Foos and Boone, 2008; Roskos-Ewoldsen et al., 2008; Palmiero et al., 2014; Addis et al., 2016). Critically, only two fMRI studies have examined the age-related changes in neural mechanisms and network interactions associated with creative cognition. In one pioneering study, Adnan et al. (2019b) used region of interest (ROI) functional connectivity analyses to examine age-related differences in functional brain networks during a divergent thinking task. The authors observed greater functional coupling of the neural patterns of default-executive network interactions in older subjects relative to their younger counterparts when both age groups exhibited equivalent in-scanner divergent creative performances (Adnan et al., 2019b). In the second study, Adnan et al. (2019a) used resting state functional connectivity (RSFC) measures to investigate age-related differences in the intrinsic brain network architecture associated with creative cognition. The authors found that the functional connectivity between the DMN, fronto-parietal ECN, and SN, as well as age-related default-executive network coupling, was associated with outside-of-scanner divergent thinking performance in older adults. The latter rs-fMRI study also identified the importance of the ventromedial prefrontal cortex as a network node with respect to functional connectivity in the creative aging brain (Adnan et al., 2019a). The results of both studies support the default-executive coupling hypothesis of aging (DECHA) proposed by Turner and Spreng (Turner and Spreng, 2015; Spreng and Turner, 2019), which posits that both the modulation of the prefrontal cortex and the suppression of the DMN, decrease with age. Moreover, the DECHA indicates the age-related alterations of brain connectivity associated with task demand and behavioral performance in older adults, with a greater reliance on cognitive-control processes which involve the prefrontal brain regions and suppression of regions of the DMN with advancing age. The DECHA further hypothesizes that there is neurocognitive flexibility between switching the use of default mode to executive process bi-directionally in younger adults. In contrast, it would become less flexible for older adults to perform the default-executive coupling and would be poorly modulated by the task context.

Researchers have suggested that functional connectivity within and between the cerebellum and cerebral cortex plays a critical role in creative cognition (Ito, 2008; Neumann et al., 2018; Ogawa et al., 2018; Sunavsky and Poppenk, 2019; Sun et al., 2019). Further, several neuroanatomical and neuroimaging studies have revealed the prominent role of the cerebellum in efficient movement, coordination, and some higher cognitive functions, including creativity (Schmahmann, 1991; Ito, 1993, 2006; Vandervert et al., 2007; Buckner, 2013). For example, in an fMRI study of functional connectivity between the cerebellum and cerebral cortex in expert musicians, Pinho et al. (2014) demonstrated increased connectivity between the fronto-parietal network, sensorimotor network (SMN), and cerebellar network (CN) during musical improvisation. In another fMRI study, Stoodley et al. (2012) demonstrated that neural activation patterns within and between cerebro-cerebellar circuits supported cognitive tasks in various domains, including sensorimotor activity, working memory, execution control, language, visuospatial, and affective processing. These findings suggest that cerebro-cerebellar interactions are critical to the coordination of sensorimotor information, control of internally focused attention, retrieval of goal-directed memories, and production of unusual thoughts (Ramnani, 2006; Honda et al., 2018; Lin et al., 2020).

As neuroimaging studies have revealed age-related functional and structural changes in the cerebellum (Raz et al., 2005; Bernard and Seidler, 2014) and indicated the important role played by cerebro-cerebellar functional connectivity in creative thinking, we examined whether and how cerebellar interactions with other cerebral networks contribute to age-related changes in creative cognition. In this rs-fMRI study, we aimed to investigate whether and how functional connected networks associated with creativity, including the DMN, fronto-parietal ECN, SN, VN, and CN, change with increasing age. To avoid the bias elicited by the a priori selection of brain regions from different age groups, we applied a group-independent component analysis (group-ICA) approach to whole-brain RSFC at the network level. ICA is a data-driven approach in which a mathematical algorithm is used to decompose the blood oxygen level dependent (BOLD) signal into spatial and temporal independent components in rs-fMRI data (McKeown et al., 1998; Beckmann and Smith, 2005; Calhoun and Adali, 2006; Cole et al., 2010; van den Heuvel and Hulshoff Pol, 2010; Bhaganagarapu et al., 2013; Rummel et al., 2013; Bi et al., 2018). This approach facilitates and improves the extraction of distinct functional connected networks. Given the unconstrained nature of the BOLD signal across younger and older participants, the estimation of group-level components in such an analysis is performed via a temporal concatenation group-ICA of data from various participants, which assumes similar spatial patterns in the brain networks across individuals (Calhoun et al., 2001; Beckmann et al., 2005; Erhardt et al., 2011; Manoliu et al., 2013). Accordingly, we then combined the group-ICA and RSFC measures to examine the effects of age on creative brain connectivity.

We assessed the creative performance of individuals using the Creative Achievement Questionnaire (CAQ), an empirically valid measure of creative performance (Carson et al., 2005). The CAQ was proposed to assess achievement across 10 domains of creativity using strong psychometric parameters, and is associated with individual differences in the neuroanatomical changes of younger adults (Jung et al., 2010). Based on previous neuroimaging studies in which the modulation of the prefrontal cortex and suppression of the DMN decreased with age (i.e., DECHA) (Turner and Spreng, 2015; Spreng and Turner, 2019), and given the critical supportive role of the cerebro-cerebellar network in higher-level cognition (Ramnani, 2006; Honda et al., 2018; Lin et al., 2020), we hypothesized that healthy older adults would exhibit altered functional connectivity in areas of the DMN, ECN, and CN associated with creative cognition. Furthermore, we hypothesized that individual differences in creative performance would modulate the effectiveness of interactions within and between creativity-associated cerebro-cerebellar networks.

## 2. Materials and Methods

### 2.1. Participants

Twenty younger adults (9 females) aged 20–29 (*M* = 23.0 years, SD = 2.23) and 34 healthy community-dwelling older adults (24 females) aged 61–80 (*M* = 63.0 years, SD = 7.65) participated in this rs-fMRI study. The older participants recruited in this study comprised mainly female participants. However, a previous study on creativity failed to identify gender-related differences in creativity (Reese et al., 2001), suggesting that the number of female participants would not affect the interpretation of our results. All of the participants were screened using a detailed self-report health questionnaire, and none had a prior history of neurological (e.g., epilepsy, traumatic head injury, or other neurological disease), or psychiatric (e.g., chronic depression) disorders. All of the participants were right-handed and exhibited normal or corrected-to-normal visual acuity during screening, and none used a hearing aid. All of the participants provided written informed consent prior to participation. The study protocol was approved by the Institutional Review Board of National Chiao Tung University, Taiwan.

### 2.2. Neuropsychological Assessment

Before undergoing MRI scanning, each participant completed the Mini-Mental State Examination (MMSE) (Folstein et al., 1975), which was designed to assess global cognitive abilities such as orientation in time and space, attention and calculation, memory, and language. We also applied a battery of neuropsychological tests from the third version of the Wechsler Adult Intelligence Scale (Wechsler, 1997a) to assess age-related and individual differences in neurocognitive function, including digit symbol coding, symbol searching, block design, picture completion, matrix reasoning, arithmetic, letter-number sequencing, forward and backward digit span, vocabulary, and similarity tests. We also applied the forward and backward spatial span, immediate and delayed facial recognition, and visual reproduction tests from the third version of the Wechsler Memory Scale (Wechsler, 1997b).

### 2.3. Creative Ability Assessment

All of the participants were instructed to complete the creative achievement questionnaire (CAQ) (Carson et al., 2005) as a measure of age-related and individual differences in creativity abilities. The CAQ is a self-reported and empirically valid measure of creative performance in which strong psychometric parameters are used to assess achievement across 10 domains of creativity: arts, music, dance, architecture, creative writing, humor, invention, scientific discovery, drama, and cooking. The CAQ score indicates whether a person's creative achievements are observable and recognized or appreciated by the public in various creativity-related domains. Following a previous behavioral study in which the CAQ score distribution appeared to be highly skewed (Silvia et al., 2009), we standardized the CAQ scores across all of the participants prior to further analysis.

### 2.4. Functional MRI Data Acquisition and Preprocessing

All of the functional and anatomical imaging was performed on a 3T Siemens MRI scanner (Magnetron Trio, Siemens, Germany) at National Yang-Ming University, Taipei, Taiwan. The participants were asked to rest with their eyes closed for 5 min during the functional imaging data acquisition.

During each session, functional images were obtained using a single shot T2*-weighted gradient echo-planer image (EPI) sequence [response time (TR)/echo time (TE) = 2,000/30 ms; flip angle = 90°]. Thirty-three contiguous axial slices were acquired, each with a slice thickness of 4 mm, a matrix of 64 × 64, and an in-plane resolution of 3.1 × 3.1 mm. Anatomical images were obtained using isotropic T1-weighted, three-dimensional (3D), ultrafast magnetization-prepared rapid acquisition with a gradient echo (MPRAGE) sequence [TR/inversion time (TI)/TE: 3,500/1,100/3.5 ms; flip angle = 7°]. One hundred and ninety-two slices with a slice thickness of 1 mm and field of view of 256 mm^2^ were acquired. All of the anatomical images were verified visually by MRI experts, and no brain abnormalities were observed.

All of the preprocessing and denoising of the acquired fMRI data were performed using the CONN toolbox (http://www.nitrc.org/projects/conn) based on MATLAB version 2019b (https://in.mathworks.com). The preprocessing of the resting-state fMRI data was performed using the standardized pre-processing pipeline in CONN for volume-based analysis (Whitfield-Gabrieli and Nieto-Castanon, 2012). The preprocessing included slice-timing correction, motion correction, realignment, and normalization. The functional data of each participant were initially realigned and unwrapped for motion estimation and correction. After re-alignment, the data were assessed and corrected for head motion artifacts. Artifact reduction tool (ART) based scrubbing was used to detect and repair bad volumes, which were defined using two measures: (1) a frame-wise displacement >0.9 mm in all directions and (2) a global mean intensity threshold >5 standard deviations from the mean intensity for the entire scan.

The functional and anatomical images were normalized separately in a standardized Montreal Neurological Institute (MNI) space and then segmented into white matter (WM), gray matter (GM), and cerebrospinal fluid (CSF) using the unified segmentation and normalization procedure (Ashburner and Friston, 2005) in SPM12 (The Wellcome Center for Human Neuroimaging, London, UK, http://www.fil.ion.ucl.ac.uk/spm/). The normalized images were then smoothed by spatial convolution with an 8-mm full-width half-maximum Gaussian kernel (FWHM). Finally, the fMRI data were bandpass-filtered between 0.008 and 0.09 Hz. The CompCor method in CONN (Behzadi et al., 2007) was also applied to interpret various principal components associated with the CSF and WM. The realignment parameters, CSF, and WM were used as confounding factors in the first-level resting state analysis (Behzadi et al., 2007).

### 2.5. Resting-State fMRI Data Analysis

The rs-fMRI data were subjected to group-ICA and assessed using functional connectivity measures.

#### 2.5.1. Group Independent Component Analysis (Group-ICA)

The group-ICA was performed using the FastICA method for the estimation of independent spatial components and group-ICA backpropagation. For this analysis, the default settings in the CONN toolbox for group-ICA were used (https://web.conn-toolbox.org/; Whitfield-Gabrieli and Nieto-Castanon, 2012). The ICA maps in our work represent measures of different resting-state brain networks and connectivity at each voxel. The approach of ICA has been widely used to identify various brain networks in resting state fMRI data. The fMRI data was processed subject-wise and the group-ICA was implemented in CONN. The group level independent component activation maps in the ICA analysis are based on group-ICA methodology used by Calhoun et al. (2001), with subject level dimension reduction, concatenation across subjects and dimensionality reduction by using group-level singular value decomposition using a fast-ICA algorithm. The number of estimated components was set at 30, and the dimensionality reduction factor was set at 64. The selection of the components identified in the ICA was based on the suggestions from the previous works which used 30 components for analyses (Calhoun et al., 2001; Li et al., 2007; Abou-Elseoud et al., 2010; Abou Elseoud et al., 2011). Furthermore, a correlational spatial match-to-template in CONN was applied to identify resting state networks (RSNs) within each component.

#### 2.5.2. Resting State Functional Connectivity (RSFC) Measures

In this study, the resting-state functional connectivity measures were based on the ROI-to-ROI analysis and seed-based connectivity measures defined in the CONN toolbox (https://web.conn-toolbox.org/).

The ROI-to-ROI analysis represents the level of functional connectivity between each pair of ROIs. The functional connectivity measures were applied to the 132 default nodes defined in the CONN atlas, which normally combines the automated anatomical labeling (AAL) atlas of 26 cerebellar regions (Tzourio-Mazoyer et al., 2002) with the FSL-based Harvard–Oxford cortical and subcortical regions. Functional connectivity measures were assessed for the resting state data of each subject, and beta values representing the averaged-Fischer transformed pairwise correlations of specified contrast were obtained for every ROI and every subject. The Student's *t*-test was used to obtain the significant statistics between these beta values and the CAQ score.

The seed based connectivity measure was based on characterizing connectivity patterns with the set of in prior seed ROIs. Moreover, the seed-based connectivity maps were obtained with the 4 DMN seeds and 26 seeds in the cerebellum defined in the CONN atlas to understand potential connectivity paths within the DMN and estimating cerebro-cerebellar connectivity measures associated with creative cognition.

The significance threshold for both functional connectivity measure and voxel-based measure was set to uncorrected voxel-wise *p* < 0.001, with a false discovery rate (FDR)-corrected cluster-wise *p* < 0.05 (Chai et al., 2014; Geissmann et al., 2018).

## 3. Results

### 3.1. Demographic and Behavioral Characteristics

All of the participants received a minimum score of 26 in the MMSE (Folstein et al., 1975), with mean scores of 29.3 and 28.7 for the younger and older participants, respectively. The older participants had fewer years of education than the younger participants (*M* = 12.79 years, SD = 3.29 and *M* = 15.45 years, SD = 1.50, respectively, *p* = 0.01). The two groups had equivalent verbal ability levels, as determined using the vocabulary test and similarity test. Regarding neuropsychological parameters, we observed inter-group differences in the processing speed (digit symbol coding test, *p* < 0.01; symbol search test, *p* < 0.01), visuospatial ability (block design test, *p* < 0.01; picture completion test. *p* < 0.05), fluid intelligence (matrix reasoning test, *p* < 0.01; arithmetic test, *p* < 0.05), working memory (letter-number sequencing, *p* < .01; digit span forward, *p* < 0.05; digit span backward, *p* < 0.01), and both immediate and delayed recall measures (visual reproduction, *p* < 0.01; immediate face recognition, *p* < 0.01; delayed face recognition, *p* < 0.01). This pattern, in which crystallized intelligence (i.e., vocabulary test) is spared while fluid intelligence (i.e., speed, executive function, and working memory) decreases with age, is typical of most samples in the literature on normal cognitive aging (Park et al., 2002; Huang et al., 2012; Fan et al., 2019). The demographics of both groups are depicted in Table 1.

**Table 1:** Demographics and Neuro-psychological results of younger and older participants.

	**Assessment used**	**Groups**	
		**Older adults**	**Younger adults**
Total	-	34	20
Age [mean (SD)]	-	63.0 (7.65) yrs.	23.0 (2.23) yrs.
Education [mean (SD)]	-	12.79 (3.29) yrs.	15.45 (1.5) yrs.
Cognitive test scores	MoCA score [mean (SD)]	24.38 (6.76)	28.95 (1.79)
	MMSE score [mean (SD)]	28.70 (1.08)	29.3 (0.80)
Creativity scores	CAQ [mean (SD)]	6.91 (21.82)	10.65 (8.76)
Memory scores	Digit-span Forward [mean (SD)]	13.64 (1.88)	15.45 (0.68)
	Digit-Span Backward [mean (SD)]	7.25 (2.23)	11.8 (2.54)
	Letter-Number sequencing [mean (SD)]	9.9 (5.21)	16.45 (3.61)

### 3.2. Resting-State fMRIs

**Figure 1:**
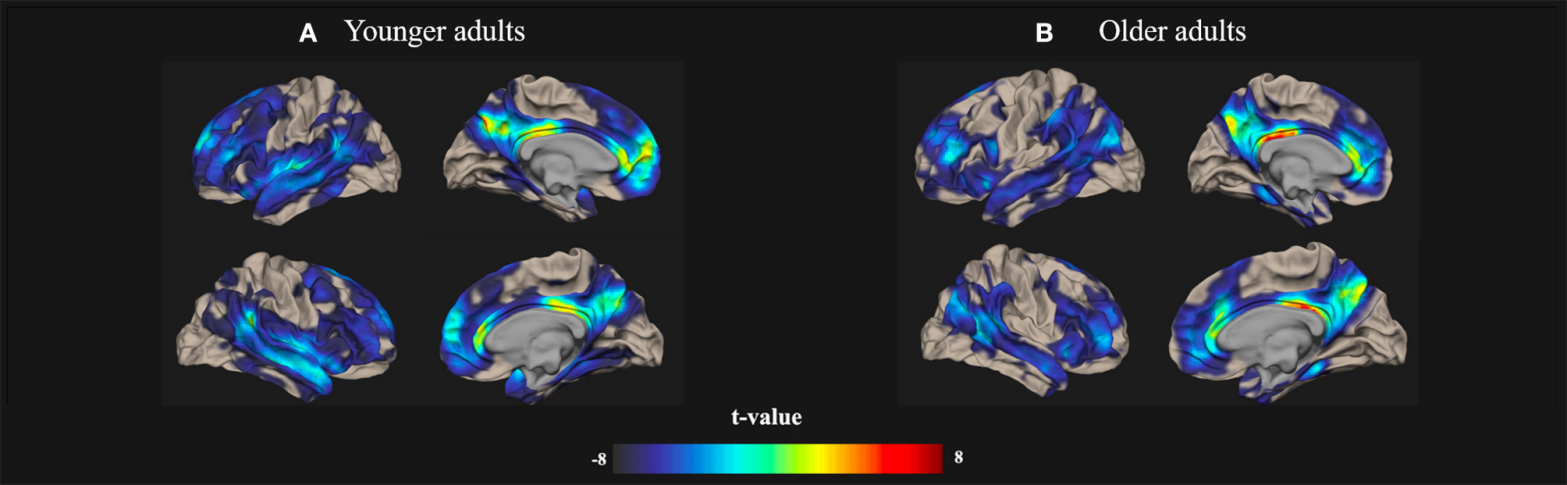
Resting-state Default-mode brain activation associated with creativity in younger and older adults. Resting-state brain activation maps depict the default-mode networks in **(A)** younger and **(B)** older adults. A significance level of voxel-level threshold *p* < 0.001 and a cluster-level threshold false discovery rate (FDR)-corrected *p* < 0.05 were applied.

All of the rs-fMRI data were analyzed using a voxel threshold *p* < 0.001 and an FDR-corrected cluster threshold *p* < 0.05. The resting-state brain activation maps of the default-mode networks in the younger and older participants are presented in Figure 1.

#### 3.2.1. Group ICA Results

**Figure 2:**
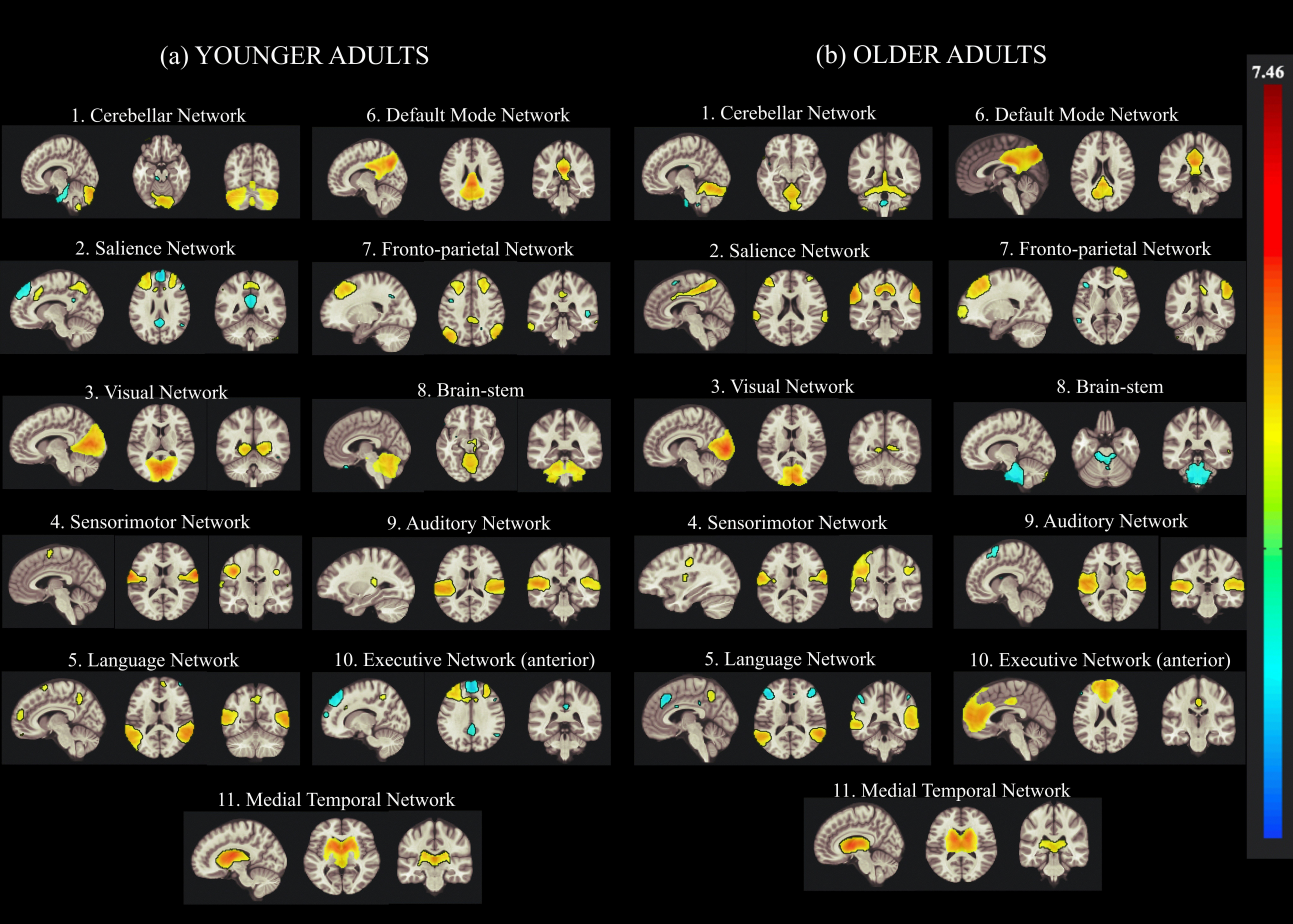
Group Independent Component Analysis (Group-ICA) of the resting-state network from concatenated datasets of **(a)** younger and **(b)** older Adults. Eleven resting-state networks were identified by applying the group-ICA approach across datasets from younger and older adults. The left side of the image represents the left side of the brain.

In this study, the group-ICA using CONN was used to identify various brain networks in resting-state fMRI data to examine age-related alterations in the functional connectivity of creative cognition. In the ICA analysis, we calculated 30 independent components with a default *z*-value threshold of 2 to identify the brain networks associated with each ICA component. The threshold of 2 is well-defined and used to characterize connections within each component in several previous resting-state fMRI studies. Eleven resting-state brain networks were obtained using this ICA approach, including the DMN, ECN, CN, VN, SN, language network (LN), fronto-parietal network (FPN), auditory network (AN), and SMR using the correlation based spatial match-to-template in CONN (Kornelsen et al., 2020). The major brain networks identified in both younger and older participants by the group-ICA are presented in Figure 2.

Table 2 presents the clustered areas of significant activation in the younger and older participants as identified by the group-ICA. No significant differences between the older and younger adults were observed in the clustered areas of activation in the DMN. In the ECN, the older adults exhibited clustered activity in the left inferior frontal gyrus, left superior parietal gyrus (SPG), and various right hemispheric regions, including the hippocampus, lingual regions, and middle frontal regions. In contrast, in the ECN of the younger adults, bilateral activity was observed in the middle temporal gyrus (MTG) and frontal gyrus (MFG). Activity was also observed in the supplementary motor area (SMA), cerebellar regions, PCC, precuneus, and left superior frontal gyrus (SFG) in younger adults.

**Table 2:** Clustered areas of significant activation during creative aging in older and younger adults identified using group independent component analysis.

**Older adults**							**Younger adults**						
**RSN**	**Brain areas**	**BA**	**x**	**y**	**z**	**Voxels**	**RSN**	**Brain areas**	**BA**	**x**	**y**	**z**	**Voxels**
	L Anterior Cingulate Cortex	32	−2	38	16	19,634		L Inferior Temporal	20	−40	0	−34	11,193
	L Inferior Temporal	37	−52	−50	−8	3,336		R Insula	47	32	20	−10	1,454
	R Inferior Temporal	37	62	−46	−18	2,697	DMN	L Inferior Frontal (orbito)	47	−34	22	−10	923
	L Inferior Frontal (orbito)	13	−30	18	−18	1,009	mPFC	R Thalamus	50	6	−8	2	392
	R Inferior Frontal (orbito)	47	34	24	−18	885		L Inferior Temporal	37	−44	−60	−8	323
	R Cerebellum Crus1	−	12	−78	−28	766		R Calcarine	17	22	−74	4	274
	L Temporal Pole (middle)	38	−54	10	−30	436							
DMN	L Cerebellum Crus2	−	−10	−76	−34	379		R Precuneus	7	10	−64	36	13,744
	L Cerebellum 9	−	−2	−48	−56	287		R Inferior Frontal (orbito)	47	38	28	−14	8,071
	R Cerebellum Crus1	−	26	−80	−34	258	DMN	R Superior Parietal	5/7	26	−82	50	3,546
mPFC	L Cerebellum Crus2	−	−28	−78	−38	256		L Superior Frontal (Medial)	8	−8	30	42	3,484
	R Cerebellum 7b	−	32	−74	−56	204		R Inferior Temporal	20	58	−20	−32	1,541
	L Cerebellum 8	−	−28	−72	−62	164	PCC	L Inferior Temporal	20	−64	−26	−22	832
	R Middle Frontal	38	52	12	−38	158		L Cerebellum 9	−	−10	−52	−44	224
	R Superior Parietal	7	22	−58	56	151		L Superior Parietal	7	−18	−56	60	144
	L Postcentral	1	−22	−30	68	143							
	L Cerebellum 45	−	−8	−62	−12	135		R Caudate	−	16	−8	14	5,714
	L Cerebellum 45	−	−14	−34	−24	125		L Postcentral	43	−52	−10	36	4,805
							DMN	R Precentral	4	50	−10	34	4,748
								L Superior Frontal	6	−18	0	64	458
	L Fusiform	37	−30	−40	−16	21,102	LP	R Primary Sensory	1	36	−46	66	205
	R Inferior Parietal	7	50	−52	54	4,719		R Postcentral	43	38	−18	−44	144
	R Superior Frontal	8	22	26	42	2,698		R Supplementary motor area	6	0	−4	68	111
	L Middle Frontal	6	−22	8	54	2,329							
	R Precuneus	7	6	−60	56	1,833		L Angular	39	−46	−58	26	12056
	L Putamen	49	−24	6	−4	1,562		L Superior Frontal	8	−12	38	50	7,117
	L Middle Temporal	21	−52	−34	−12	1,006		L Middle Temporal	21	−56	−10	−24	2,975
DMN	R Supplementary motor area	6	10	18	64	937		R Cerebellum 9R	−	10	−58	−52	2,039
	R Middle Cingulate Cortex	23	2	−18	32	655		R Posterior Cingulate Cortex	23	0	−52	28	1,629
PCC	L Anterior Cingulate Cortex	24	−6	36	−4	590		L Precuneus	7	−6	−76	46	1,475
	R Middle Temporal	21	52	−34	−10	563	ECN	R Middle Temporal	21	56	−8	−24	1,347
	L Inferior Temporal	20	−50	2	−38	375		R Inferior Parietal	40	56	−38	48	698
	R Inferior Frontal (triangular)	45	48	22	4	359		L Middle Frontal	6	−36	12	52	431
	R Inferior Temporal	20	54	−4	−42	298		R Supplementary motor area	6	8	−12	56	322
	L Cerebellum Crus1	−	−44	−70	−32	238		R Postcentral	43	22	−46	60	304
	R Putamen	49	24	10	0	136		R Cerebellum Crus1	−	24	−78	−34	245
	L Superior Frontal	6	−28	−10	70	134		R Middle Frontal	8	36	16	40	214
	R Superior Temporal	39	58	−46	18	12,078							
	L Middle Temporal	21	−54	−34	−4	10,415		R Middle Temporal		54	−58	16	6,322
	L Middle Frontal	46	−40	40	14	10,108		L Superior Temporal		−62	−56	14	4,983
	R Precuneus	7	6	−58	50	3,077	DMN	R Middle Frontal		42	0	56	1,416
	L Inferior Parietal	40	−46	−44	48	2,266	Angular Gyrus	R Precuneus		8	−50	46	931
	R Middle Frontal	10	40	46	6	1,628		R Superior Frontal (medial)		8	52	22	439
	R Precentral	6	44	6	42	1,435		L Cerebellum (Crus1)		−22	−78	−32	342
	R Superior Frontal (Medial)	9	8	52	30	1,327		L Precentral		−40	−4	44	264
	R Inferior Parietal	40	52	−36	50	1,314		R Insula		40	14	−10	125
	L Middle Frontal	6	−42	8	50	965		R Inferior Frontal (orbito)		48	30	−4	97
DMN	L Cerebellum Crus2	−	−22	−80	−36	950							
LP	R Supplementary motor area	6	14	18	66	876							
	L Inferior Temporal	37	−56	−46	−16	665							
	R Rectus	11	4	48	−24	614							
	R Cerebellum Crus2	NA	22	−76	−36	570							
	L Middle Cingulate Cortex	23	−2	−34	36	553							
	R Cerebellum Crus2	−	44	−68	−44	553							
	L Cerebellum 9	−	−4	−50	−52	525							
	R Inferior Temporal	37	52	−52	−16	514							
	L Hippocampus	30	−18	−36	2	167							
	L Cerebellum Crus2	−	−42	−62	−42	119							
	L Superior Frontal	6	−16	−10	76	116							
	L Inferior Frontal (triangular)	44	−42	20	16	39,179							
	R Lingual	18	10	−82	−6	462							
ECN	R Middle Frontal	46	46	12	56	296							
	R Para Hippocampal	34	26	−40	−4	210							
	L Superior Parietal	7	−20	−56	72	186							

*Peaks were identified using Montreal Neurological Institute (MNI) coordinates. All results were obtained at a FWE-corrected significance threshold of p < 0.05. An independent component analysis was used to obtain the different resting state networks, with a minimum cluster size of 8 mm. RSN, resting state networks; BA, Brodmann area; DMN, default mode network, mPFC, pre-frontal cortex (medial); PCC, posterior cingulate cortex; LP, lateral parietal; ECN, executive control network; L/R, Left/Right; x, y, and z, coordinates in MNI space for peak voxels in activated regions; Vol, voxels in the peak activated regions*.

In the CN, the older participants exhibited activation in the cerebellum and the anterior and middle cingulate cortex. Furthermore, the older adults exhibited activity in the precentral gyrus, insula, and supplementary motor area. In contrast, the younger adults exhibited activity in the inferior and superior frontal gyrus (IFG and SFG), putamen, and fusiform. The latter group also exhibited greater activity in the frontal regions of the brain relative to the cerebellar and temporal-occipital regions. The cerebellar cluster identified using the group-ICA is depicted in **Table 4**.

#### 3.2.2. RSFC of the Creative Brain in Younger and Older Adults

**Figure 3:**
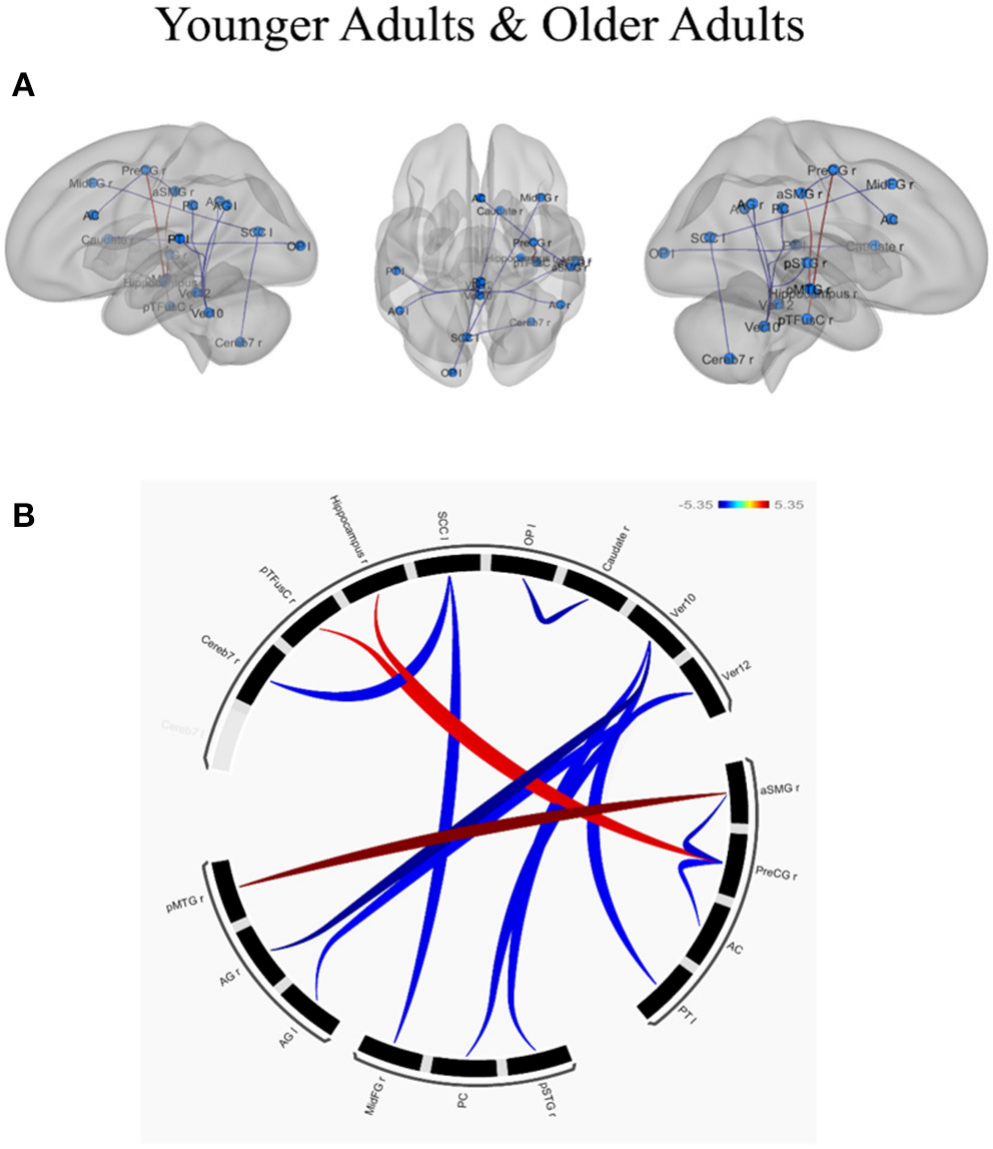
Effect of creative performance (measured by Creative Achievement Questionnaire, CAQ) on resting-state functional connectivity represented using **(A)** 3D-Glass brain and **(B)** Connectogram. **(A)** 3D-Glass brain: Each node is represented as a sphere (in cyan color); the positive and negative connections are represented in red and blue color, respectively. **(B)** Connectogram: Each node is coded with black color; the positive and negative connections are represented in red and blue color, respectively. The results were thresholded using a false discovery rate (FDR)-corrected *p* < 0.05 for multiple testing correction (corrected by individual differences in educational level). OP l, left occipital pole; Caudate r, right caudate; Ver12, vermis lobule 12; Ver10, vermis lobule 10; aSMG r, right supramarginal gyrus (anterior); PreCG r, left precentral gyrus; AC, anterior cingulate cortex; PT l, left planum temporale; pSTGr, right superior temporal gyrus (posterior); PC, posterior cingulate cortex; MidFGr, right middle frontal gyrus; AG l, left angular gyrus; AG r, right angular gyrus; pMTGr, right middle temporal gyrus (posterior); Cereb 7r, right cerebellum lobule 7; pTFusC r, right temporal fusiform cortex (posterior); Hippocampus r, right hippocampus; SCC l, left supra-calcarine cortex.

ROI-to-ROI analysis was conducted to examine whether and how individual differences in creative ability, as measured by CAQ scores (controlled by individual differences in educational level), influenced the functional connected networks in younger and older adults. The results of the age-related functional connectivity within and between various brain networks associated with creative performance are presented in Table 3. The effect of creative performance, measured by CAQ on resting-state functional connectivity is depicted with 3D-Glass brain in Figure 3A and Connectogram in Figure 3B.

**Table 3:** Effect of CAQ scores on resting-state functional connectivity in younger adults.

**ROI 1**	**BA**	**ROI 2**	**BA**	**Beta**	**T(17)**	**p-FDR**
Right precentral gyrus	6	Right Hippocampus	30	0.02	4.35	0.02477
Right precentral gyrus	6	Right Temporal fusiform cortex (posterior)	37	0.02	4.23	0.02477
Right precentral gyrus	6	Anterior Cingulate gyrus	23/31	-0.02	-4.03	0.02866
Right precentral gyrus	6	Right Supra marginal gyrus (anterior)	40	-0.02	-4.53	0.02477
Right middle temporal gyrus (posterior)	21	Right Supra marginal gyrus (anterior)	40	0.02	5.35	0.00689
Right Angular gyrus	41/42	Cerebellum (vermis 10)	-	-0.02	-5.08	0.01204
Left Supracalcarine cortex	17	Right middle frontal gyrus	6	-0.02	-4.23	0.03855
Left Supracalcarine cortex	17	Cerebellum (crus 7)	-	-0.01	-4.21	0.03855
Left Occipital pole	19	Right caudate	8	-0.02	-4.79	0.02234
Cerebellum (Vermis 12)	-	Posterior Cingulate gyrus	23/31	-0.01	-4.27	0.04796
Cerebellum (Vermis 12)	-	Left Angular gyrus	41/42	-0.01	-4.11	0.04796
Cerebellum (vermis 10)	-	Left Planum Temporale	22	-0.02	-4.15	0.02900
Cerebellum (vermis 10)	-	Right Superior Temporal gyrus (posterior)	38	-0.02	-4.18	0.02900

*CAQ, Creative Achievement Questionnaire; ROI, region of interest; BA, Brodmann area; p-FDR, false discovery rate-corrected p-value*.

In younger adults, when the right medial temporal gyrus within the DMN (coordinates: *x* = 61, *y* = −22, *z* = −12) was selected as a seed ROI, higher connectivity with the right supra marginal gyrus (*t* = 5.35; p-FDR = 0.0068). When ROIs in the right angular gyrus were used as the seed ROIs (*x* = 51, *y* = −51, *z* = 32), lower connectivity with the cerebellum was observed (*t* = −5.08; p-FDR = 0.0120). When ROIs in the right precentral gyrus were used as a seed (*x* = 34, *y* = −10, *z* = 50) higher connectivity with the right hippocampus (*t* = 4.35; p-FDR = 0.0247) and right temporal fusiform cortex (*t* = 4.23; p-FDR = 0.0247) whereas lower connectivity with the anterior cingulate cortex (*t* = −4.03; p-FDR = 0.0286) and right supra marginal gyrus (*t* = −4.53; p-FDR = 0.0286) were observed. Within the VN, the left occipital pole exhibited lower connectivity with the right caudate (*t* = −4.79; p-FDR = 0.0223), the left supra calcarine cortex exhibited lower connectivity with the right middle frontal gyrus (*t* = −4.23; p-FDR = 0.0385) and the cerebellum (*t* = −4.21; p-FDR =0.0385). Within the CN, when the cerebellum was used as the seed ROI (*x* = 33, *y* = −63, *z* = −48), lower connectivity exhibited with the posterior cingulate gyrus (*t* = −4.27; p-FDR =0.0479), left angular gyrus (t = -4.11; p-FDR = 0.0479), left planum temporale (*t* = −4.15; p-FDR = 0.0290) and right superior temporal gyrus (*t* = −4.18; p-FDR = 0.0290). In contrast, the older adults exhibited no connectivity with the effect of CAQ when the ROI-to-ROI analysis was applied. The results have been depicted in Table 3.

Regarding network-specific interactions affected by CAQ scores, we observed greater functional connectivity between the SMN and the dorsal attention network (*t* = −4.30; p-FDR < 0.05) in the younger adults, but did not observe this effect of CAQ scores on connectivity in the older adults. Regarding cerebro-cerebellar interactions, the anterior CN exhibited connectivity with the dorsal attention network (*T* = 2.66; *p* < 0.05) and the posterior CN exhibited connectivity with the VN (*T* = −2.16; *p* < 0.05) in the younger adults. In the older adults, the anterior CN exhibited connections with the DMN (*T* = 3.20; *p* < 0.05) and SN (*T* = −2.35; *p* < 0.05), whereas the posterior CN exhibited connections with the dorsal attention network (*T* = −2.63; *p* < 0.05).

The voxel-wise analysis was performed on the cerebellar regions to understand the functional connections between the cerebellum and the cerebral regions during creative cognition processes. The significant clustered cerebellar areas in younger adults are presented in Table 4. The older adults did not show any functional connections from the cerebellum to the cerebral regions which correlated with the CAQ score.

**Table 4:** Resting-state correlations with clusters throughout the brain in younger adults with 26 cerebellar seed ROIs.

**Seed ROI**	**Brain areas**	**BA**	**x**	**y**	**z**	**voxels**
**YOUNGER ADULTS**						
R Cerebellum (45)	Left cingulate gyrus	23	−6	−48	34	100
R Cerebellum (7)	Right lingual gyrus	−	16	−88	0	558
	Left Insula	13	−40	−2	14	127
	Right precentral gyrus	1	56	−12	34	95
L Cerebellum (8)	Right precentral gyrus	6	50	−2	34	161
R Cerebellum (9)	Left middle frontal gyrus	6	−34	6	58	208
	Left lentiform nucleus	−	−12	0	−2	204
R Cerebellum (10)	Left lingual gyrus	18	−6	−82	−8	274
	Left middle occipital gyrus	19	−34	−86	16	88
Vermis (12)	Left cingulate gyrus	23	−12	−44	28	817
	Left medial frontal gyrus	−	−16	52	12	607
	Right Inferior parietal lobule	39	46	−60	38	416
	Right medial frontal gyrus	10	6	50	−8	272
	Left superior frontal gyrus	8	−10	34	52	142
	Right superior frontal gyrus	6	20	26	60	119
	Left middle frontal gyrus	8	−34	18	46	91
Vermis (3)	Left supramarginal gyrus	−	−64	−48	32	126
	Right medial frontal gyrus	9	8	48	22	94
Vermis (9)	Left parahippocampal gyrus	37	−22	−42	−10	127
Vermis (10)	Right Inferior parietal lobule	40	54	−42	22	278
	Left superior temporal gyrus	22	−62	−44	14	89

*Peaks were identified using the MNI coordinates. All results were obtained with the threshold significance set to an uncorrected p < 0.001 at a voxel-wise level, with the false discovery rate (FDR)-corrected p < 0.05 at the cluster-wise level. L/R, Left/Right; X, Y, and Z, coordinates in Montreal Neurological Institute (MNI) space for peak voxels in activated regions; Vol, voxels in peak activated regions; seed ROI, seed region of interest; BA, Brodmann area*.

Regarding region-specific interactions within the cerebellum affected by the CAQ scores, the results of the functional connectivity analysis revealed that younger adults showed positive correlations between the CAQ score and higher functional connectivity within the cerebellum. In older adults, however, showed positive correlations between the CAQ score and lower functional connectivity in the cerebellum. The resting-state positive and negative correlations within the cerebellum in younger and older adults are depicted in Table 5.

**Table 5:** Resting-state positive and negative correlations within the cerebellum in younger adults and older adults.

**Younger adults**				**Older adults**			
**ROI 1**	**ROI 2**	**r**	**p**	**ROI 1**	**ROI 2**	**r**	**p**
Left cerebellum (crus 1)	Vermis 7	0.46	0.0455^*^	Left cerebellum (crus 1)	Right cerebellum (crus 3)	0.397	0.0498^*^
Left cerebellum (crus 6)	Vermis 7	0.458	0.0398^*^	Vermis 45	Vermis 3	0.467	0.0009^**^
Right cerebellum (crus 45)	Vermis 45	0.476	0.0345^*^	Right cerebellum (crus 1)	Right cerebellum (crus 45)	0.412	0.0468^*^
Right cerebellum (crus 6)	Right cerebellum (crus 10)	0.467	0.0478^*^	Vermis 6	Vermis 3	0.364	0.0341^*^
Vermis 6	Vermis 7	0.461	0.0399^*^	Right cerebellum (crus 8)	Right cerebellum (crus 10)	0.402	0.0459^*^
Vermis 45	Vermis 6	0.546	0.0421^*^	Right cerebellum (crus 45)	Right cerebellum (crus 6)	0.350	0.0497^*^
Right cerebellum (crus 1)	Vermis 6	0.474	0.0498^*^	Left cerebellum (crus 2)	Vermis 12	-0.351	0.0323^*^
Right cerebellum (crus 2)	Vermis 6	0.491	0.0468^*^	Left cerebellum (crus 10)	Vermis 12	-0.382	0.0487^*^
Right cerebellum (crus 3)	Left cerebellum (crus 8)	0.473	0.0421^*^	Right cerebellum (crus 45)	Left cerebellum (crus 7)	-0.407	0.0457^*^
Right cerebellum (crus 8)	Vermis 9	-0.595	0.0005^**^	Right cerebellum (crus 7)	Vermis 6	-0.41	0.0364^*^
Right cerebellum (crus 10)	Vermis 9	-0.586	0.0007^**^	Left cerebellum (crus 7)	Right cerebellum (crus 10)	-0.382	0.0477^*^
Left cerebellum (crus 7)	Vermis 10	-0.491	0.0482^*^	Left cerebellum (crus 8)	Vermis 6	-0.353	0.0498^*^
Left cerebellum (crus 7)	Vermis 9	-0.526	0.0386^*^	Right cerebellum (crus 6)	Left cerebellum (crus 7)	-0.349	0.0399^*^
Left cerebellum (crus 9)	Vermis 10	-0.523	0.0398^*^	Right cerebellum (crus 6)	Right cerebellum (crus 10)	-0.398	0.0317^*^
				Left cerebellum (crus 7)	Vermis 8	-0.439	0.0477^*^
				Left cerebellum (crus 7)	Vermis 6	-0.427	0.0487^*^

ROI, Region of interest; r, Pearson's correlation coefficient; p, Significance value. All results were obtained with the threshold significance set to an uncorrected p < 0.001 at a voxel-wise level, with the false discovery rate (FDR)-corrected p < 0.05 at the cluster-wise level.

* p < 0.05;

** *p < 0.001*.

## 4. Discussion

Using a group-ICA and RSFC approach, this rs-fMRI study identified age-related differences in functional connectivity within the default-executive and cerebro-cerebellar network interactions associated with creativity. The group-ICA approach identified 11 major brain networks across age groups, which reflected the age-invariant resting-state networks. Compared with the older participants, the younger participants exhibited more specific and widespread dorsal network and SMN connectivity within and between the DMN, VN, AN, fronto-parietal network, and CN. These observations suggest the occurrence of age-specific changes in the functional connected network, particularly in the default-executive network and cerebro-cerebellar network. In the younger adults, the connections identified between the cerebellum and para-cingulate cortex identified regions critical to pre-potent response inhibition that were not seen in older adults. Our connectivity data further elucidate the critical roles of the cerebellum and cerebro-cerebellar connectivity (particularly the DMN, fronto-parietal network, VN, SN, and dorsal attention network) in the creative brain with age. Further, our findings are consistent with the DECHA and provide novel evidence supporting cerebro-cerebellar network interactions related to creative cognition in older adults. Taken together, our results suggest changes to creative cognitive processes in the aging brain.

Several studies have explored the potential link of RSFC between various brain networks with creative cognition. A functional link has been identified between creative cognition and the DMN, especially the PCC, medial prefrontal cortex, and lateral parietal regions (Takeuchi et al., 2011; Beaty et al., 2015, 2018a). Behavioral measures have been used to link resting-state connections between the DMN and ECN with creative cognition (Beaty et al., 2018b). In this rs-fMRI study, we observed significant positive connections with DMN regions and other DMN and posterior regions in the older adults, and these positive connections were correlated significantly with the creative cognition scores. In the younger adults, the DMN exhibited significant positive connections only with frontal brain regions. The more negative connectivity in older adults relative to their younger counterparts suggests the existence of age-related changes in functional connected networks within and between the DMN. Similarly, when seed ECN regions were used, significant connections with the posterior and frontal regions were observed in the older adults, whereas only connections with the frontal regions were observed in the younger adults. When seed VN (i.e., temporo-occipital regions) regions were used, both the older and the younger adults exhibited positive significant connections with the creative cognition scores. Our observations of functional connectivity in the DMN and ECN are consistent with the DECHA, and studies related to that hypothesis also observed a beneficial shift in the neural architecture related to creative cognition in older adults (Adnan et al., 2019a,b). The older adults in our study also exhibited stronger cerebellar connectivity between posterior regions compared with the frontal regions, regardless of creative score modulation. In contrast, the younger adults exhibited cerebro-cerebellar connectivity within and between regions of the CN, fronto-parietal network, DMN, VN, AN, and SMN. The relatively strong connectivity in the posterior regions (vs. frontal regions) of the older adults suggests that a shift in functional connectivity patterns with age could partially support the concept of a posterior-anterior shift in aging (Davis et al., 2008; Zhang et al., 2017).

In our rs-fMRI study, we further examined the critical role of the cerebellum and its distributed connectivity with various regions of the brain associated with creative cognition. Although the cerebellum has traditionally been understood to contribute motor control (Ito, 2000; Ivry et al., 2002; Bastian, 2006), recent studies have suggested that this brain region likely contributes to various complex cognitive tasks (Diedrichsen et al., 2019; Schmahmann, 2019). The cerebellum comprises 69 billion neurons, whereas the cerebral cortex contains only 16 billion neurons (Lent et al., 2012; Lin et al., 2020; Sereno et al., 2020). Consequently, the cerebellum contributes to complex circuitous processes such as sensorimotor function, cognitive functions, and autonomic tasks (Baumann et al., 2015; Schmahmann et al., 2019). The cerebellum has also been shown to contribute to cognitive abilities such as spatial reasoning, language, and working memory (Schmahmann and Sherman, 1998; Ito, 2008; Stoodley and Schmahmann, 2009; Moberget and Ivry, 2016). In addition to anatomical findings, previous rs-fMRI studies have suggested that higher-level cognitive and non-motor functions may be supported by cerebro-cerebellar connections (Habas et al., 2009; Krienen and Buckner, 2009; O'Reilly et al., 2010). Together with evidence from previous rs-fMRI studies that suggested the distribution of CN participation in sensorimotor and higher cognitive functions, our findings suggest that changes in functional connectivity patterns within and between the cerebro-cerebellar circuitry may be critical to understanding the process of complex cognition, including creativity (Akshoomoff et al., 1997; Imamizu et al., 2003; Lin et al., 2020; Sereno et al., 2020).

In our study, CAQ scores exhibited a significant association with RSFC. In older adults, higher connectivity was observed in the superior frontal and posterior regions of the brain, which exhibited significant correlations with individual CAQ scores. In summary, CAQ scores were correlated with regions involving creative cognition in the older participating adults. In contrast, the younger adults exhibited higher connectivity in most of the frontal regions, and this was significantly correlated with CAQ scores. In the cingulate cortex, the older adults exhibited higher connectivity, whereas the younger adults exhibited lower connectivity. Both the older and younger adults also exhibited lower RSFC in these regions, which were significantly correlated with the CAQ scores. We also analyzed functional connectivity in the angular gyrus, which serves as a hub for higher cognitive functions (Fjell et al., 2015) related to episodic and semantic memory, language, and visual, attention, and social cognition (Vincent et al., 2006; Seghier et al., 2010; Price et al., 2015). In the older adults, higher functional connectivity was observed in posterior regions of the angular gyrus (e.g., planum polare, central and frontal operculum), and these correlated with the CAQ scores. In the younger adults, correlations were observed between frontal regions of the angular gyrus (inferior, middle, and superior) and CAQ scores, which indicated higher connectivity within the angular gyrus and particularly the frontal regions. In the fusiform gyrus resting state network, older adults with higher CAQ scores exhibited stronger connectivity in the frontal and parietal regions, and this effect was observed from the posterior to the anterior part of the aging brain. Finally, we explored the contributions of various cerebellar areas to creativity. Although the older adults exhibited smaller increases in cerebellar connectivity than the younger adults, the former group exhibited a greater number of links related to creativity. The observations of significant correlations between the CAQ score and the cerebellum in both older and younger adults emphasize the role of cerebellar resting-state connectivity in creativity.

Some potential limitations of this study must be noted. First, the behavioral data were collected using a standard questionnaire. Future investigations could examine the task-based creative potential of individual participants. Second, the analysis of resting-state connectivity between creative regions in the younger and older participants did not consider individual differences in the CAQ scores or the high and low CAQ scores. Third, the data were collected from a sample of Taiwanese adults, so the generalizability of the results may be limited. Given the previous studies suggested the effects of age on cognitive tasks may be modulated by cultural values (Park and Huang, 2010; Na et al., 2017), future studies should examine creative cognition in a cross-cultural sample. A few other limitations in our study were the lower sample size and limited statistical power due to 132 ROIs and 11 RSNs. Despite having these limitations, the results have been consistent with prior rs-fMRI creativity works and match with the earlier neuroimaging results and this study identified brain regions and networks that can predict creative styles and achievements and differences in these aspects between younger and older adults.

In conclusion, this combined group-ICA and rs-fMRI study provides network neuroscience based evidence that age-related changes in functional connectivity in the default-executive and cerebro-cerebellar networks modulate the process of creative cognition. Our findings lay a foundation for further studies of how cerebro-cerebellar network interactions may contribute to age-related and individual differences in complex cognitive processes.

## Data Availability Statement

The raw data supporting the conclusions of this article will be made available by the authors, without undue reservation.

## Ethics Statement

The studies involving human participants were reviewed and approved by The Institutional Review Board of National Chiao Tung University, Taiwan. The patients/participants provided their written informed consent to participate in this study.

## Author Contributions

AP, DM, and C-MH designed the project. AP and C-MH performed the experiment, analyzed the data, and wrote the manuscript. All authors have read and approved the final version of the manuscript.

## Conflict of Interest

The authors declare that the research was conducted in the absence of any commercial or financial relationships that could be construed as a potential conflict of interest.
